# A broad and potent neutralization epitope in SARS-related coronaviruses

**DOI:** 10.1073/pnas.2205784119

**Published:** 2022-06-29

**Authors:** Meng Yuan, Xueyong Zhu, Wan-ting He, Panpan Zhou, Chengzi I. Kaku, Tazio Capozzola, Connie Y. Zhu, Xinye Yu, Hejun Liu, Wenli Yu, Yuanzi Hua, Henry Tien, Linghang Peng, Ge Song, Christopher A. Cottrell, William R. Schief, David Nemazee, Laura M. Walker, Raiees Andrabi, Dennis R. Burton, Ian A. Wilson

**Affiliations:** ^a^Department of Integrative Structural and Computational Biology, The Scripps Research Institute, La Jolla, CA 92037;; ^b^Department of Immunology and Microbiology, The Scripps Research Institute, La Jolla, CA 92037;; ^c^IAVI Neutralizing Antibody Center, The Scripps Research Institute, La Jolla, CA 92037;; ^d^Consortium for HIV/AIDS Vaccine Development (CHAVD), The Scripps Research Institute, La Jolla, CA 92037;; ^e^Adimab, LLC, Lebanon, NH 03766;; ^f^Ragon Institute of Massachusetts General Hospital, Massachusetts Institute of Technology and Harvard University, Cambridge, MA 02139;; ^g^Adagio Therapeutics, Inc., Waltham, MA 02451;; ^h^The Skaggs Institute for Chemical Biology, The Scripps Research Institute, La Jolla, CA 92037

**Keywords:** SARS-CoV-2, variants of concern, cross-neutralizing antibody, conserved epitope, X-ray crystallography

## Abstract

Many neutralizing antibodies (nAbs) elicited to ancestral severe acute respiratory syndrome coronavirus 2 (SARS-CoV-2) through natural infection and vaccination have reduced effectiveness to SARS-CoV-2 variants. Here, we show that therapeutic antibody ADG20 is able to neutralize SARS-CoV-2 variants of concern (VOCs) including Omicron (B.1.1.529) as well as other SARS-related coronaviruses. We delineate the structural basis of this relatively escape-resistant epitope that extends from one end of the receptor binding site (RBS) into the highly conserved CR3022 site. ADG20 can then benefit from high potency through direct competition with ACE2 in the more variable RBS and interaction with the more highly conserved CR3022 site. Importantly, antibodies that are able to target this site generally neutralize a broad range of VOCs, albeit with reduced potency against Omicron. Thus, this conserved and vulnerable site can be exploited for the design of universal vaccines and therapeutic antibodies.

## Significance

Since the beginning of the COVID-19 pandemic, many severe acute respiratory syndrome coronavirus 2 (SARS-CoV-2) variants have emerged that are resistant to varying extents to neutralizing antibody responses induced by current vaccines and natural infection, especially the recent Omicron variants. Neutralizing potency and breadth for an antibody are often somewhat mutually exclusive. Here, we delineate the molecular interaction between a therapeutic antibody (ADG20) and SARS-CoV-2 receptor-binding domain (RBD) by X-ray crystallography and characterize its binding epitope. We show that this site is targeted by a few rare antibodies that have both potency and breadth. These findings provide insights into the design of more universal vaccines and broad therapeutic antibodies, which are pressingly needed.

Severe acute respiratory syndrome coronavirus 2 (SARS-CoV-2) vaccines, based on the ancestral virus strain (1, 2), confer protective immunity and greatly decrease the incidence of infection, disease severity, and hospitalization from COVID-19. Many SARS-CoV-2 variants have emerged, and the designated variants of concern (VOCs), especially the recent Omicron variants, as well as some variants of interest, are much more resistant to neutralizing antibody (nAb) responses induced by current vaccines (3–8). A vaccine that is highly protective against current SARS-CoV-2 VOCs could potentially provide broader protection against future emerging variants and possibly other sarbecoviruses. However, neutralizing potency and breadth are often somewhat mutually exclusive; the most highly potent nAbs target the ACE2 receptor binding site (RBS) of the spike (S) protein, but most SARS-CoV-2 VOCs have mutations in the RBS that reduce nAb binding and neutralization. Broad binding antibodies, such CR3022, that target other epitopes on the receptor binding domain (RBD) usually have lower neutralization potency (9). Here, we identify a site of vulnerability on the RBD of the SARS-CoV-2 S protein that is targeted by a few diverse antibodies. Importantly, such antibodies, as exemplified by ADG20, compete with receptor binding, exhibit high neutralizing potency and show broad activity to VOCs, including Omicron, to a conserved region that is present also on other SARS-related coronaviruses (CoVs) including SARS-CoV-1, WIV1, and SHC014.

## Results

### Crystal Structure of ADG20 in Complex with SARS-CoV-2 RBD.

Some of the authors previously developed mAb ADG20 as an extended half-life version of potent-and-broad human antibody ADG-2 (10, 11). ADG20 and ADG-2 share the same antigen-binding fragment (Fab) domain with a few amino acid changes in the fragment crystallizable region (Fc region) (11). ADG20 and ADG-2 neutralize a broad spectrum of SARS-related CoVs including SARS-CoV-2, SARS-CoV-1, WIV-1, and SHC014 with high potency (fifty percent maximal inhibitory concentration [IC_50_] ranging from 1 to 30 ng/mL against authentic viruses), and confer outstanding protection in mouse models chalenged with SARS-CoV-1 and SARS-CoV-2 (10). ADG20 is now in phase II/III trials for COVID-19 treatment and prevention (12, 13). A low-resolution cryo-electron microscopy structure (∼6 Å) of ADG20 Fab was previously reported in complex with the SARS-CoV-2 S protein (10).

**Fig. 1.**
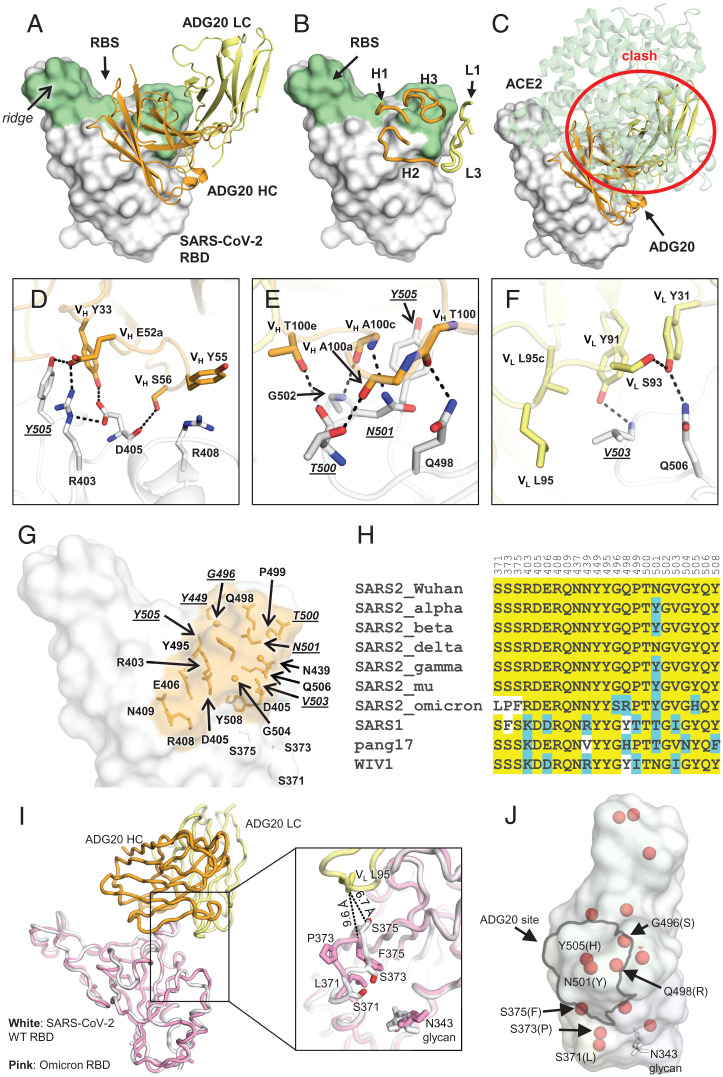
Crystal structure of ADG20 in complex with SARS-CoV-2 RBD. The heavy and light chains of ADG20 are shown in orange and yellow, respectively. SARS-CoV-2 RBD is shown in white. ADG20 epitope residues and RBS residues are defined by residues with surface area buried by ADG20 and ACE2 of >0 Å^2^, respectively, and calculated based on the ADG20/RBD crystal structure in this study and an RBD/ACE2 complex structure (PDB 6M0J) (54) by PISA (50). (*A*) Structure of ADG20 Fab in complex with SARS-CoV-2 RBD. The RBS is shown in green. For clarity, the constant domains of ADG20 Fab were omitted. (*B*) ADG20 CDRs that interact with SARS-CoV-2 are shown as tubes. (*C*) The ACE2/RBD complex structure is superimposed on the ADG20/RBD complex. ADG20 would clash with ACE2 (green) if bound to RBD simultaneously (indicated by red circle). (*D* to *F*) Detailed interactions between ADG20 (D) CDRs H1 and H2, (*E*) CDR H3, and (*F*) light chain with SARS-CoV-2 RBD. Hydrogen bonds and salt bridges are represented by black dashed lines. Epitope residues involved in ACE2 binding are underlined and italicized. Kabat numbering is used for antibody residues throughout this paper. (*G*) Epitope residues of ADG20 are highlighted in orange, with side chains shown as sticks and Cα of glycines as spheres. (*H*) Sequence alignment of RBD residues in a subset of SARS-like viruses where we also generated PSV neutralization data. Residues within the ADG20 epitope as well as those in the 371 to 375 loop are aligned. Conserved residues among SARS-related CoVs are highlighted with a yellow background, while similar residues are in a cyan background (amino acids scoring greater than or equal to 0 in the BLOSUM62 alignment score matrix (55) were counted as similar here). (*I*) Structural comparison between the ADG20-bound WT SARS-CoV-2 RBD and Omicron RBD (BA.1 sublineage). Crystal structure of ADG20 (heavy chain: orange, light chain: yellow) in complex with WT SARS-CoV-2 RBD (white) is from this study (PDB 7U2D). The superimposed Omicron RBD (pink) is extracted from a previous structure in complex with two other Fabs (PDB 7QNW) (19). (*J*) The RBD is represented by a transparent white surface. Mutated residues in the Omicron variant (BA.1 sublineage) are shown as red spheres. The ADG20 epitope is highlighted by black solid lines. The following four residues in the ADG20 epitope are mutated in the Omicron variant: G496S, Q498R, N501Y, and Y505H.

Here, we determined a crystal structure of ADG20 Fab in complex with the wild-type (WT) SARS-CoV-2 RBD to 2.75-Å resolution to decipher the atomic details of the antibody–antigen interactions and the molecular features of this site of vulnerability (*SI Appendix*, Tables S1 and S2). ADG20 targets one corner of the RBD that is distant from the protruding ridge region (Fig. 1 *A*–*C*) through complementarity determining regions (CDRs) H1, H2, H3, L1, and L3 (Fig. 1*B*). The epitope is distinct from any of the antibody classes I to V recently analyzed in Yin et al. (14) (*SI Appendix*, Fig. S1). The buried surface areas (BSAs) of SARS-CoV-2 RBD conferred by the heavy and light chains of ADG20 are 488 and 204 Å^2^, respectively. The epitope of ADG20 overlaps with the RBS (Fig. 1 *A* and *B*), and binding of the antibody would clash with ACE2 binding to the RBD (Fig. 1*C*). The epitope of ADG20 is only accessible when RBD is in the up conformation (*SI Appendix*, Fig. S2). CDRs H1 and H2 of ADG20 participate in a network of interactions with the RBD (Fig. 1*D*), where V_H_ E52a forms a hydrogen bond and salt bridge with Y505 and R403, respectively. R403 is further stabilized by D405, which hydrogen bonds with V_H_ S56 and V_H_ Y33. V_H_ Y33 in turn stacks with Y505. V_H_ Y55 interacts with R408 through a cation–π interaction (Fig. 1*D*). CDR H3 forms five hydrogen bonds with the RBD (Fig. 1*E*). The light chain of ADG20 is also involved in RBD recognition, where V_L_ Y91, L95, and L95c form a hydrophobic pocket to accommodate V503. V_L_ Y91 and Y31 hydrogen bond with V503 and Q506, respectively (Fig. 1*F*). G504 is also involved in the interaction with ADG20 (Fig. 1*G*). In our escape mutation study, RBD-G504D emerged in a second passage of authentic SARS-CoV-2 in the presence of ADG20 and exhibited full escape (*SI Appendix*, Fig. S3), consistent with our previous finding where G504D abrogates binding of ADG-2 to the SARS-CoV-2 RBD (10), illustrating the importance of this interaction.

### ADG20 Broadly Neutralizes SARS-CoV-2 Variants and Other SARS-Like CoVs.

**Fig. 2.**
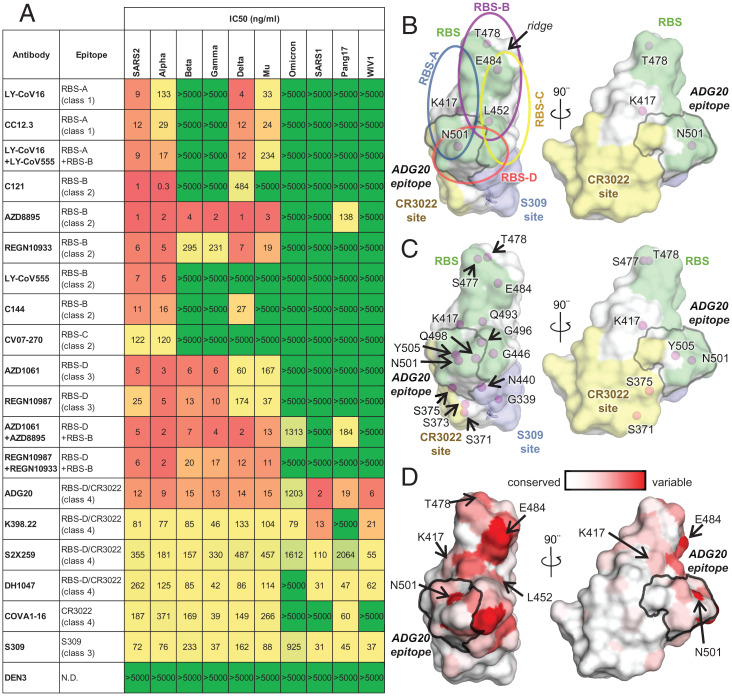
Neutralization of antibodies targeting different epitopes against SARS-CoV-2 variants and other SARS-related CoVs. (*A*) Neutralization of antibodies against pseudotyped SARS-CoV-2 variants and other SARS-related CoVs. Ancestral SARS-CoV-2 is shown as “SARS2”. Epitope classes are as defined in ref. 15. Antibody epitopes that include residues in both RBS-D and CR3022 sites are shown as “RBS-D/CR3022”. Corresponding categories (class 1 to 4) that were classified in ref. 56 are shown in brackets. (*B*, *C*) A map of RBD epitopes and mutated residues in SARS-CoV-2 VOCs. The RBS, CR3022 site, and S309 site are shown in green, yellow, and lavender, respectively. The ADG20 epitope is highlighted with a gray outline. Mutated residues in SARS-CoV-2 VOCs Alpha, Beta, Gamma, and Delta are labeled in *B*, and those mutated in the VOC Omicron are labeled in *C*. The N343 glycan is shown as sticks. Four RBS subsites, namely, RBS-A, -B, -C, and -D, are indicated by circles in *B* but omitted in *C* for clarity. Antibodies targeting the RBS-A/class 1 epitope are mainly encoded by IGHV3-53/3-66 genes. This class of antibodies is generally sensitive to mutations K417N/T in Beta, Gamma, and Omicron (*SI Appendix*, Fig. S7*A*). Many RBS-B/class 2 (including another major class of antibodies that are encoded by IGHV1-2 and share structural convergence ([*SI Appendix*, Fig. S7*B*]) and RBS-C antibodies are often sensitive to E484 mutations in the ridge region of the RBD. L452 is located on one edge of the RBS and interacts with many RBS-B and RBS-C antibodies (*SI Appendix*, Fig. S7*C*). N501 is located in the RBS-A and RBS-D epitopes, but N501Y, the only mutated RBD residue in the Alpha variant, is often tolerated by RBS-A and RBS-D antibodies (*SI Appendix*, Fig. S4). The Alpha variant not unexpectedly has the least immune escape among all VOCs. (*D*) Sequence variation of 14 SARS-related CoVs (including ancestral SARS-CoV-2 and variants Alpha, Beta, Gamma, Delta, Mu, Omicron, and SARS-CoV-1, BM4831, BtKY72, pang17, RaTG13, RsSHC014, and WIV1) mapped onto an RBD structure (from white [low] to red [high]) The ADG20 epitope is highlighted with a gray outline. Mutated residues in SARS-CoV-2 VOCs are indicated by arrows.

The ADG20 epitope residues are generally conserved among SARS-CoV-2 and its variants, as well as other SARS-related CoVs including SARS-CoV-1 (Fig. 1 *G* and *H*). Notably, unlike some major classes of RBD-targeting nAbs (e.g., IGHV3-53 and IGHV1-2 antibodies), which are sensitive to mutations in Beta and Gamma variants (15), all of the ADG20 epitope residues are conserved among VOCs Alpha, Beta, Gamma, and Delta, except for N501Y (Fig. 1*H*), which only minimally affects the interaction with ADG20 (*SI Appendix*, Fig. S4). The SARS-CoV-2 Omicron BA.1 variant fully escapes neutralization by 14 out of 18 tested mAbs and mixture pairs. By contrast, ADG20 retains neutralization activity against Omicron (IC_50_ = 1.2 μg/mL), although with ∼100-fold reduction compared to ancestral SARS-CoV-2 (IC_50_ = 12 ng/mL) (Fig. 2*A*). The neutralization activity of ADG20 against Omicron is comparable with the Evusheld mixture of antibodies AZD1061 + AZD8895 (IC_50_ = 1.3 μg/mL) but is much more potent against SARS-CoV-1 and other sarbecoviruses (IC_50_ = 2 to 19 ng/mL) in the panel of viruses tested (Fig. 2*A*). ADG20 and a previously discovered antibody, JMB2002, that has completed a Phase 1 clinical trial (14, 16), target opposite sides of the RBD (*SI Appendix*, Fig. S5), but both retain neutralization activity against the Omicron variant. Sotrovimab (derived from S309), a clinically authorized antibody for emergency use, is less affected by the Omicron variant, but because its starting potency against the WT is lower, its absolute IC_50_ (0.9 μg/mL) against Omicron is similar to ADG20. Thus, ADG20 is one of the most potent nAbs among all tested antibodies against a panel of viruses (Fig. 2*A*). Our observations are consistent with previously reported neutralization results performed with pseudotyped and authentic viruses (17–19). Several ADG20-epitope contact residues from SARS-CoV-2 differ in SARS-CoV-1, pang17, and WIV1 (Fig. 1*H*) but apparently can be accommodated by ADG20 (*SI Appendix*, Fig. S4).

### Crystal Structure of ADI-55688, the Parental Antibody of ADG20.

ADG20 is an affinity-matured progeny of ADI-55688, a broad RBD-targeting monoclonal antibody isolated from a SARS-CoV-1-convalescent donor (10, 20). Like ADG20, ADI-55688 cross-reacts with RBDs of SARS-CoV-2 and SARS-CoV-1 and neutralizes both viruses (10). ADI-55688 differs from ADG20 by only five amino acids, with three located in the heavy chain and two in the light chain (*SI Appendix*, Fig. S6*A*). These mutated residues in ADG20 confer a nearly 200-fold improved binding affinity and a 100-fold increased neutralizing activity against SARS-CoV-2 compared to ADI-55688 (10). We also determined a crystal structure of ADI-55688 Fab in complex with SARS-CoV-2 RBD at 2.85 Å and compared it with the ADG20/RBD structure. These two antibodies target the same epitope through a near-identical binding approach (*SI Appendix*, Fig. S6*B*). The V_H_ S52a substitution by glutamic acid in ADG20 leads to a salt bridge with RBD-R403 (*SI Appendix*, Fig. S6*C*), and the V_H_ W100b mutation from valine increases the interaction with V_L_ H34 and V_H_ F96. These substitutions appear to stabilize the conformation of the light and heavy chain CDRs (*SI Appendix*, Fig. S6*C*) and result in an improved off-rate (10).

### Structural Basis of SARS-CoV-2 VOC Escape from Antibodies.

RBD is the major target of nAbs against SARS-CoV-2 (21, 22). To understand the differential effects of binding and neutralization by ADG20 compared to other RBD antibodies, we first mapped all of the VOC mutations in Alpha, Beta, Gamma, and Delta onto the RBD epitope classes. Only five mutated residues, namely, K417, L452, T478, E484, and N501, are located in the RBD in these VOCs and are distributed throughout the RBS and cover all four RBS epitopes (Fig. 2*B*). In contrast, 15 residues are mutated in Omicron BA.1 RBD (G339D, S371L, S373P, S375F, K417N, N440K, G446S, S477N, T478K, E484A, Q493R, G496S, Q498R, N501Y, and Y505H), where 8 of these 15 residues are directly involved in ACE2 binding (Fig. 2*C*). The RBS is the most variable site among SARS-related viruses (Fig. 2*D*), but this high variation is tolerated by the receptor. In contrast, none of the mutations of VOCs Alpha, Beta, Gamma, and Delta are found in the relatively conserved CR3022 and S309 sites, although 4 of the 15 Omicron mutations are located in either the CR3022 (371 and 375) or S309 (339 and 440) sites (Fig. 2 *B* and *C*). Previously, we classified the RBD-targeting antibodies into six sites, as follows: RBS sites RBS-A, -B, -C, and -D; CR3022 site; and S309 site (Fig. 2*B*) (15). These antibodies are often encoded by different germline genes with different sensitivities to VOC mutations (15). We show here the impact of the Omicron mutations on all of these sites (Fig. 2*C*). The S371L mutation in Omicron BA.1 is in close proximity to the N343 glycan (Fig. 1*I*) which may induce a concerted effect on the local conformation and dynamics. Mutations in the 371 to 375 region (S371L, S373P, and S375F) of the Omicron variant induce a backbone shift away from the ADG20 paratope (Fig. 1 *I* and *J*) and decrease ADG20 neutralization (18). In fact, the S371L mutation reduced the neutralization potency of most tested class 1, 3, and 4 antibodies, suggesting that such effects may result from backbone shifts in the loop containing residue 371 in WT versus Omicron variants (18). In addition, four mutations in Omicron BA.1 reside within the ADG20 epitope (Fig. 1 *G*–*J*), but single mutations of these four residues minimally alter ADG20 neutralization (18).

### A Common Epitope Targeted by Broad and Potent nAbs.

**Fig. 3.**
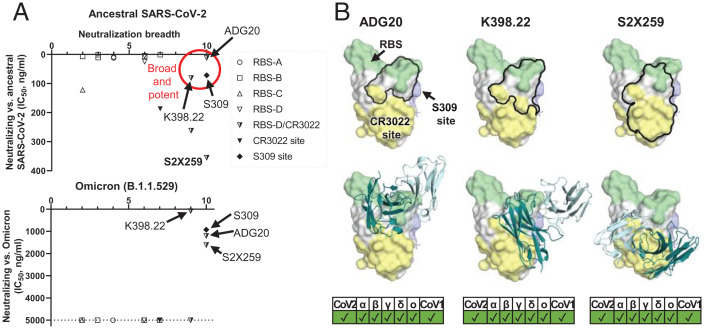
A common region on the RBD targeted by potent-and-broad nAbs against SARS-CoV-2. (*A*) A scatter plot of antibody neutralization potency (IC_50_, ng/mL) against (*Top*) ancestral SARS-CoV-2 and (*Bottom*) Omicron vs. neutralization breadth, defined by the number of SARS-related CoV strains neutralized in this study (10 strains in total, including ancestral SARS-CoV-2, Alpha, Beta, Delta, Gamma, Mu, Omicron, SARS-CoV-1, pang17, and WIV1). (*B*) All of the potent-and-broad nAbs target a region that spans from one end to the RBS (green) to the proximal CR3022 site (yellow). Epitopes of each antibody are outlined by black lines (*Top*), where the variable domains of ADG20, K398.22, and S2X259 are shown in cartoon representation with heavy chains in deep teal and light chains in light cyan (*Bottom*). Epitopes and the RBS are defined by residues with BSA of >0 Å^2^ as calculated by PISA (50) using structures of ADG20 (this study), K398.22 (26), S2X259 (PDB 7RAL) (57), CR3022 (PDB 6W41) (9), S309 (PDB 6WPS) (24), and ACE2 (PDB 6M0J) (54). SARS-CoV-2 variants as well as SARS-CoV-1 can be neutralized by these antibodies, as shown at the *Bottom* of the panel.

To gain further insights into ADG20 protection against SARS-CoV-2 and other sarbecoviruses, we further analyzed the binding and neutralizing activities of representative antibodies targeting the different class of RBD epitopes, including some of the antibodies authorized for COVID-19 therapeutic prevention and/or treatment against SARS-CoV-2 and VOCs (*SI Appendix*, Fig. S4). We then compared the neutralization potency and breadth of each antibody versus ADG20 in a potency vs. breadth plot (Fig. 3*A*). Although most RBS antibodies are potent against the ancestral SARS-CoV-2, they are sensitive to VOC mutations, suggesting that potency is usually associated with a tradeoff in breadth. Most tested RBS-A, -B, and -C antibodies can be escaped by at least one VOC (Fig. 2*A*) due to the mutated residues being largely located in the RBS (Fig. 2 *B* and *C*). In contrast, the CR3022 site is much more conserved than the RBS (Fig. 2*C*) (9, 15, 21). CR3022-site antibody COVA1-16 is a broadly neutralizing antibody (23) but is less potent than most RBS antibodies and loses neutralization capabilities against Omicron. Other CR3022-site targeting antibodies, e.g., S304 and CR3022, have also been reported to have broad binding breadth but low or no neutralization potency to SARS-CoV-2 (9, 24, 25). Binding breadth but limited neutralization potency for an antibody may seem a paradox in many cases but may be due to insufficient affinity, relative inaccessibility of the epitope on the S, or inability to directly compete with the ACE2 receptor.

Notwithstanding, a particularly noteworthy epitope is targeted by a few potent-and-broad antibodies including ADG20 (Figs. 2*A* and 3). All four tested antibodies (ADG20, DH1047, S2X259, and K398.22) exhibited high (<20 ng/mL) to moderate (40 to 500 ng/mL) potency to CoVs that include SARS-CoV-2, Alpha, Beta, Gamma, and Delta VOCs and other SARS-related CoVs including SARS-CoV-1, pang17, and WIV1, with ADG20 exhibiting high potency to all of these CoVs (Fig. 2*A*). DH1047 and ADI-55688, which was evolved in vitro to produce ADG20, were both isolated from a SARS-CoV-1 convalescent donor. This epitope seems infrequently targeted by the SARS-CoV-2 antibody repertoire, probably explaining why mAbs directed to this site are rarely isolated and described in the literature. Importantly, unlike most potently neutralizing antibodies targeting other antigenic sites on the RBD, ADG20, S2X259, and K398.22 also demonstrated neutralization activity to Omicron (Fig. 2*A*). These antibodies target a site on the RBD spanning the RBS-D and CR3022 sites (Fig. 3) and are encoded by various germline genes with distinct CDR H3 sequences (*SI Appendix*, Table S3). DH1047 also targets the RBS-D/CR3022 site and neutralizes most SARS-CoV-2 VOCs and SARS-CoV-1 but did not show neutralization activity against Omicron at the highest concentration of antibody used (5,000 ng/mL) (*SI Appendix*, Fig. S8). This effect may be a consequence of its relatively low neutralization against the ancestral SARS-CoV-2 (IC_50_ = 262 ng/mL) (Fig. 2*A*) and slight differences in the key contact residues. This result is consistent with a previous study where DH1047 exhibited low neutralization activity (IC_50_ of ∼10,000 ng/mL) against Omicron pseudovirus (PSV) (18). Antibodies targeting the other side of the RBS-D site (*SI Appendix*, Fig. S8), namely, AZD1061 and REGN10987, are not able to neutralize the Omicron BA.1 variant or other tested SARS-related CoVs such as SARS-CoV-1, pang17, and WIV1, possibly due to the relatively low conservation of this subepitope (Fig. 2 *A* and *C* and *SI Appendix*, Fig. S8*A*). LY-CoV1404 also binds to the same side of RBS-D (*SI Appendix*, Fig. S5) and was reported to neutralize Omicron but not bind SARS-CoV-1 (27). Thus, antibodies like ADG20 take advantage of the properties of two sites: targeting the RBS region that confers direct competition with receptor binding and strong potency and, on the other hand, targeting the conserved CR3022 site that imparts breadth against not only SARS-CoV-2 VOCs but also other SARS-related CoVs.

## Discussion

The Omicron variant has spread globally at an unprecedented rate with the highest level of immune evasion so far in all observed VOCs (3–5). Omicron is resistant, or has reduced effectiveness, to most current authorized therapeutic monoclonal antibodies as well as mixtures, including LY-CoV555, LY-CoV016, REGN10933, REGN10987, and BRII-196. However, neutralization by S309 or its derivative Sotrovimab/VIR-7831 was only reduced by 3- to 10-fold against Omicron BA.1 (5, 17, 18, 28–30) compared to 40- to 100-fold reduction for ADG-2/ADG20 against Omicron pseudotyped virus here (Fig. 2*A*) and in another study (18). Importantly, using authentic viruses, only a 20-fold reduction of ADG20 neutralization against Omicron was observed compared to Delta and was the most potent among all the tested therapeutic antibodies (17). Recently, a sublineage of the Omicron variant, BA.2 (B.1.1.529.2), has emerged. Compared to the Omicron BA.1 sublineage, BA.2 has one reversion (S446G) and three additional mutations (T376A, D405N, and R408S), as well as an alternative mutation (S371F instead of S371L in BA.1) in the RBD (*SI Appendix*, Fig. S9). BA.2 evades ADG20 and other antibodies targeting this site (e.g., S2X259) (31, 32), which may be attributable to an effect that is promulgated through differences in the interaction between residue 371 and the N343 glycan.

**Fig. 4.**
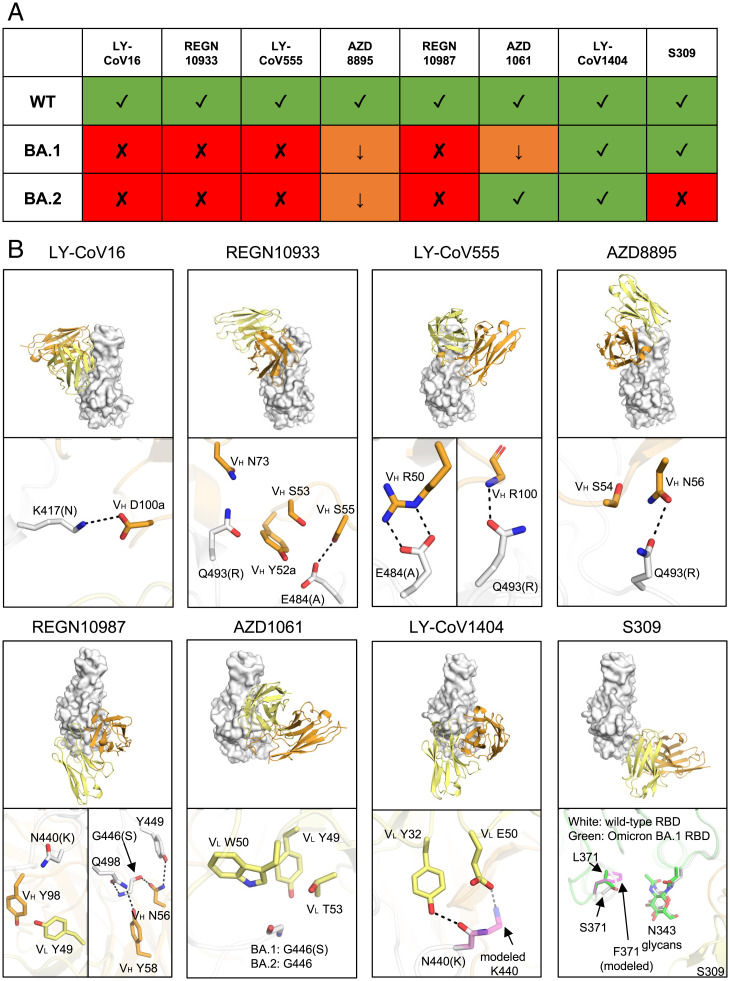
Mechanisms of escape or resistance to therapeutic antibodies against SARS-CoV-2. (*A*) Neutralization activity of the therapeutic antibodies shown are taken from refs. 18, 30, 31. Variants that retain neutralization activity are represented by green “✓” symbols, while variants with reduced or abolished neutralization are indicated by orange “↓” and red “✘” symbols, respectively. (*B*) Structural explanation of escape mechanisms of the Omicron BA.1 and BA.2 sublineages. RBDs are represented by white surfaces, while the heavy and light chains of the antibodies are in orange and yellow cartoon representation. Hydrogen bonds and salt bridges are shown as black dashed lines. For clarity, only the variable domains of the antibodies are shown. Orientations of the RBDs are the same for all the eight antibodies, as follows: LY-CoV016 (PDB 7C01), REGN10933 (6XDG), LY-CoV555 (7KMG), AZD8895 (7L7D), REGN10987 (6XDG), AZD1061 (7L7E), LY-CoV1404 (7MMO), and S309 (7R6W). The structure of S309 in complex with Omicron BA.1 RBD (green, PDB 7TN0) was superimposed onto the S309/WT-RBD structure for comparison. Mutations S371F for S309-bound RBD as well as N440K for LY-CoV1404-bound RBD are modeled and represented by transparent magenta sticks. Kabat numbering is assigned to all antibody residues.

Several therapeutic antibodies have been developed against SARS-CoV-2, including REGEN-COV (REGN10933/casirivimab plus REGN10987/imdevimab), sotrovimab (S309/Vir-7831), bamlanivimab/LY-CoV555 plus etesevimab/LY-CoV016, Evusheld (tixagevimab/AZD8895 plus cilgavimab/AZD1061), and bebtelovimab (LY-CoV1404) (33). Within these five antibody therapeutics (eight antibodies total), bebtelovimab retained activity against both Omicron BA.1 and BA.2, whereas Evusheld potently neutralized BA.2 but not BA.1 (Fig. 4*A*) (18, 30, 31). LY-CoV016 binds to RBS-A, where the Omicron mutation K417N results in the loss of a salt bridge (Fig. 4*B*) and abolishes neutralization. REGN10933, LY-CoV555, and AZD8895 bind to RBS-B, where mutations E484A and Q493R caused a loss of polar interactions and possible clashes (Fig. 4*B*), resulting in the reduction of neutralization potency. REGN10987, AZD1061, and LY-CoV1404 target the RBS-D site. While N440K may clash with REGN10987 and reduce its neutralization, this mutation may result in the loss of a hydrogen bond and gain of a salt bridge with LY-CoV1404. The G446S mutation, which only occurs in Omicron BA.1 but not in BA.2, may also clash with AZD1061 (Fig. 4*B*), which could explain the BA.1-specific escape against this antibody (Fig. 4*A*). Sotrovimab targets the S309 site involving interactions with the N-glycans at N343. The S371F mutation in the BA.2 sublineage may result in a less favorable position of the N343 glycan and lead to a reduction in neutralization potency (18, 30, 31). During the revision process of this manuscript, new variants BA.2.12.1, BA.4, and BA.5 have emerged with additional mutations L452Q/R and F486V (https://www.outbreak.info/) (*SI Appendix*, Fig. S10*A*). F486 stacks with multiple aromatic rings of AZD8895 (*SI Appendix*, Fig. S10*B*), where F486V exhibited 121- to 149-fold reduction in its neutralization (34). L452 interacts with AZD1061 (*SI Appendix*, Fig. S10*B*), while L452R exhibited little or no reduction to AZD1061 neutralization (34). LY-CoV1404 is distant from F486 or L452 (*SI Appendix*, Fig. S10*C*), where its neutralization showed no reduction by these mutations (27). Consistent with the structural observation, a very recent study showed reduced or abolished neutralization of AZD8895 against the pseudotyped viruses of BA.2.12.1, BA.4, and BA.5, with little or no effect on LY-CoV1404 (35).

In summary, we identify a vulnerable site on the SARS-CoV-2 RBD that antibodies can target and mitigate against the mutations found in VOCs. These relatively rare antibodies to date extend their binding interactions from the RBS, where they can directly compete with ACE2 binding, to the most highly conserved site on the RBD, where their overall footprint on the RBD confers both neutralization potency and breadth. We also recently reported a class of antibodies that possess long CDRs H3 with a YYDRxG motif that target the highly conserved CR3022 site. Such antibodies can block ACE2 without direct binding to the RBS (36), a few of which exhibit impressive breadth and potency to a wide range of SARS-CoV-2 VOCs and sarbecoviruses (36,37). The neutralization effectiveness of antibodies generated during infection or vaccination have been substantially reduced by emerging SARS-CoV-2 variants (3–5, 38–40). The VOC mutations until Omicron have been largely confined to the RBS, and yet these same mutations do not adversely affect receptor binding and viral entry in host cells. Universal vaccines or antibody therapeutics that are insensitive or less susceptible to SARS-CoV-2 mutations are urgently needed to protect against the continuous antigenic drift of the virus (41). Several universal vaccine designs have been proposed and tested, including mosaic nanoparticles conjugated with various RBDs (42) and chimeric S mRNA-based vaccines (43). Notwithstanding, such vulnerable sites in the RBD may currently be a desirable target for universal vaccine design and for antibody therapeutics.

## Materials and Methods

### Expression and Purification of IgGs and Fabs.

The heavy and light chains were cloned into phCMV3. The plasmids were transiently cotransfected into ExpiCHO cells at a ratio of 2:1 (heavy chain:light chain) using ExpiFectamine CHO Reagent (Thermo Fisher Scientific) according to the manufacturer’s instructions. The supernatant was collected at 10 d posttransfection. The IgGs and Fabs were purified with a CaptureSelect CH1-XL Affinity Matrix (Thermo Fisher Scientific) followed by size exclusion chromatography.

### Crystallization and Structure Determination.

The expression and purification of the SARS-CoV-2 S RBD for crystallization were as described previously (9). Briefly, the RBD (residues 333 to 529) of the SARS-CoV-2 S protein (GenBank: QHD43416.1) was cloned into a customized pFastBac vector (44) and fused with an N-terminal gp67 signal peptide and C-terminal His_6_ tag (9). A recombinant bacmid DNA was generated using the Bac-to-Bac system (Life Technologies). Baculovirus was generated by transfecting purified bacmid DNA into Sf9 cells using FuGENE HD (Promega) and subsequently used to infect suspension cultures of High Five cells (Life Technologies) at an multiplicity of infection of 5 to 10. Infected High Five cells were incubated at 28 °C with shaking at 110 rpm for 72 h for protein expression. The supernatant was then concentrated using a 10-kDa molecular weight cutoff Centramate cassette (Pall Corporation). The RBD protein was purified by Ni-nitriloacetic acid, followed by size exclusion chromatography, and buffer exchanged into 20 mM Tris⋅HCl (pH 7.4) and 150 mM NaCl.

ADG20/RBD and ADI-55688/RBD complexes were formed by mixing each of the protein components at an equimolar ratio and incubating overnight at 4 °C. The protein complex was adjusted to 12 mg/mL and screened for crystallization using the 384 conditions of the Joint Center for Structural Genomics (JCSG) Core Suite (Qiagen) on our robotic CrystalMation system (Rigaku) at Scripps Research. Crystallization trials were set up by the vapor diffusion method in sitting drops containing 0.1 μL of protein and 0.1 μL of a reservoir solution. For the ADG20/RBD complex, optimized crystals were then grown in drops containing 0.1 M sodium citrate (pH 4.16) and 1.45 M ammonium sulfate at 20 °C. Crystals appeared on day 7, were harvested on day 15 by soaking in reservoir solution supplemented with 15% (vol/vol) ethylene glycol, and then flash cooled and stored in liquid nitrogen until data collection. Diffraction data were collected at cryogenic temperature (100 K) at beamline 23-ID-B of the Advanced Photon Source (APS) at Argonne National Labs. For the ADI-55688/RBD complex, optimized crystals were then grown in drops containing 0.08 M sodium acetate (pH 3.8), 1.6 M ammonium sulfate, and 20% (vol/vol) glycerol at 20 °C. Crystals appeared on day 7, were harvested on day 10 by soaking in reservoir solution supplemented with 20% (vol/vol) ethylene glycol, and then flash cooled and stored in liquid nitrogen until data collection. Diffraction data were collected at cryogenic temperature (100 K) at the Stanford Synchrotron Radiation Lightsource (SSRL) on Scripps/Stanford beamline 12-1. Diffraction data were processed with HKL2000 (45). Structures were solved by molecular replacement with PHASER (46) using models of the RBD and COVA2-39 derived from Protein Data Bank (PDB) 7JMP (47). Iterative model building and refinement were carried out in COOT (48) and PHENIX (49), respectively. Epitope and paratope residues, as well as their interactions, were identified by accessing Proteins, Interfaces, Structures and Assemblies (PISA) at the European Bioinformatics Institute (https://www.ebi.ac.uk/pdbe/prot_int/pistart.html) (50).

### Biolayer Interferometry Binding Assay.

RBD proteins for the biolayer interferometry (BLI) binding assay were expressed in human cells. RBDs were cloned into phCMV3 vector and fused with a C-terminal His_6_ tag. The plasmids were transiently transfected into Expi293F cells using ExpiFectamine 293 reagent (Thermo Fisher Scientific) according to the manufacturer’s instructions. The supernatant was collected at 7 d posttransfection. The His_6_-tagged proteins were then purified with Ni Sepharose Excel protein purification resin (Cytiva) followed by size exclusion chromatography. Omicron RBD was purchased from ACROBiosystems Inc.

The BLI assays were performed using an Octet Red instrument (FortéBio) as described previously (9). To measure the binding kinetics of anti-SARS-CoV-2 IgGs and RBDs, the IgGs were diluted with kinetic buffer (1× phosphate-buffered saline [pH 7.4], 0.01% bovine serum albumin and 0.002% Tween 20) into 15 µg/mL. The IgGs were then loaded onto anti-human IgG Fc (AHC) biosensors and interacted with a fivefold gradient dilution (500 nM to 20 nM) of SARS-CoV-2 RBDs and 500 nM of RBDs of SARS-related CoVs. The assay consisted of the following steps: 1) baseline, 1 min with 1× kinetic buffer; 2) loading, 90 s with IgGs; 3) wash, 15 s wash of unbound IgGs with 1× kinetic buffer; 4) baseline, 1 min with 1× kinetic buffer; 5) association, 90 s with RBDs; and 6) dissociation, 90 s with 1× kinetic buffer. For estimating the dissociation constant (*K*_D_), a 1:1 binding model was used.

### PSV Neutralization Assay.

PSV preparation and assays were performed as previously described with minor modifications (51). Pseudovirions were generated by cotransfection of HEK293T cells with MLV-gag/pol (Addgene #14887) and MLV-Luciferase (Addgene #170575) plasmids and SARS-CoV-2 S WT or variants with an 18-amino acid truncation at the C terminus. Supernatants containing pseudotyped virus were collected 48 h after transfection and frozen at −80 °C for long-term storage. The PSV neutralizing assay was carried out as follows: 25-μL serial dilutions of purified antibodies in Dulbecco’s modified Eagle medium with 10% heat-inactivated fetal bovine serum, 4 mM L-glutamine, and 1% penicillin–streptomycin were incubated with 25 µL PSV at 37 °C for 1 h in 96-well half-well plates (Corning, 3688). After incubation, 10,000 HeLa-hACE2 cells were added to the mixture with 20 µg/mL dextran (Sigma, 93556-1G) to enhance infectivity. At 48 h postincubation, the supernatant was aspirated, and HeLa-hACE2 cells were then lysed in luciferase lysis buffer (25 mM Glegly [pH 7.8], 15 mM MgSO_4_, 4 mM EGTA, and 1% Triton X-100). Bright-Glo (Promega, E2620) was added to the mixture following the manufacturer’s instruction, and luciferase expression was read using a luminometer. Samples were tested in duplicate, and assays were repeated at least twice for confirmation. Fifty percent maximal inhibitory concentrations (IC_50_s), the concentrations required to inhibit infection by 50% compared to the controls, were calculated using the dose response–inhibition model with the five-parameter Hill slope equation in GraphPad Prism 7 (GraphPad Software).

### SARS-CoV-2 Escape Assay.

Escape assays in the presence of ADG20 were performed using authentic SARS-CoV-2, as previously described (52). Briefly, 10^5^ Median Tissue Culture Infectious Dose (TCID_50_) of an early, Wuhan-like SARS-CoV-2 strain (2019-nCoV/Italy/INMI1) was added to serial dilutions of ADG20 IgG ranging from 4.9 ng/mL to 10,000 ng/mL The mixture was incubated for 1 h at 37 °C with 5% CO_2_ before being added to a 24-well plate coated in a subconfluent Vero E6 cell monolayer. The plate was incubated for 5 d at 37 °C and 5% CO_2_ and examined for signs of cytopathic effect (CPE). The viral sample at the lowest mAb dilution exhibiting complete CPE was used as the stock for the subsequent passage. Virus was also passaged in the absence of antibody to control for tissue-culture adaptations that arise independent of antibody pressure. At each passage, both the no-antibody control and virus under selection pressure with ADG20 were harvested; propagated in 25-cm^2^ flasks; and aliquoted at −80 °C for RNA extraction, RT-PCR, and sequencing.

### Next-Generation Sequencing (NGS) of Virus Escape Variants.

NGS of the SARS-CoV-2 S gene was performed at Science Park. Viral RNA was reverse transcribed and prepared for NGS using the Swift Amplicon SARS-CoV-2 research panel (Swift Biosciences), following the manufacturer’s instructions. Libraries were quantified by qPCR using the Kapa Lib Quant Kit (Roche Diagnostics), pooled at equimolar concentrations, and sequenced using the Illumina MiSeq system (2 × 250-bp paired-end mode). Sequences were trimmed using Cuadapt v2.8, and consensus sequences, defined as a sequence present in >50% of reads, were generated via de novo sequence construction using MegaHit (53).

## Supplementary Material

Supplementary File

## Data Availability

The X-ray coordinates and structure factors have been deposited in the Research Collaboratory for Structural Bioinformatics (RCSB) Protein Data Bank under accession codes 7U2D and 7U2E.
