# Supplementary material for: A broad and potent neutralization epitope in SARS-related coronaviruses
Source: Proc Natl Acad Sci U S A. 2022 Jun 29;119(29):e2205784119. doi: 10.1073/pnas.2205784119 (PMC9304036; doi:10.1073/pnas.2205784119)
Supplement: Supplementary File [file pnas.2205784119.sapp.pdf]

Supplementary Information for

A broad and potent neutralization epitope in SARS-related coronaviruses

Meng Yuan[a,1], Xueyong Zhu[a,1], Wan-ting He[b,c,d], Panpan Zhou[b,c,d], Chengzi I. Kaku[e], Tazio Capozzola[b], Connie Y. Zhu[a], Xinye Yu[a], Hejun Liu[a], Wenli Yu[a], Yuanzi Hua[a], Henry Tien[a], Linghang Peng[b], Ge Song[b], Christopher A. Cottrell[b,c,d], William R. Schief[b,c,d,g], David Nemazee[b], Laura M. Walker[e,f], Raiees Andrabi[b,c,d], Dennis R. Burton[b,c,d,g], Ian A. Wilson[a,c,d,h,*]

[a] Department of Integrative Structural and Computational Biology, The Scripps Research Institute, La Jolla, CA 92037, USA

[b] Department of Immunology and Microbiology, The Scripps Research Institute, La Jolla, CA 92037, USA

[c] IAVI Neutralizing Antibody Center, The Scripps Research Institute, La Jolla, CA 92037, USA

[d] Consortium for HIV/AIDS Vaccine Development (CHAVD), The Scripps Research Institute, La Jolla, CA 92037, USA

[e] Adimab, LLC, Lebanon, NH 03766, USA

[f] Adagio Therapeutics, Inc., Waltham, MA 02451, USA

[g] Ragon Institute of Massachusetts General Hospital, Massachusetts Institute of Technology, and Harvard University, Cambridge, MA 02139, USA

[h] The Skaggs Institute for Chemical Biology, The Scripps Research Institute, La Jolla, CA, 92037, USA

[1] M.Y. and X.Z. contributed equally to this work

*** Corresponding author:** Ian A. Wilson. **Email:** wilson@scripps.edu

This PDF file includes:

Figures S1 to S10
Tables S1 to S3
SI References

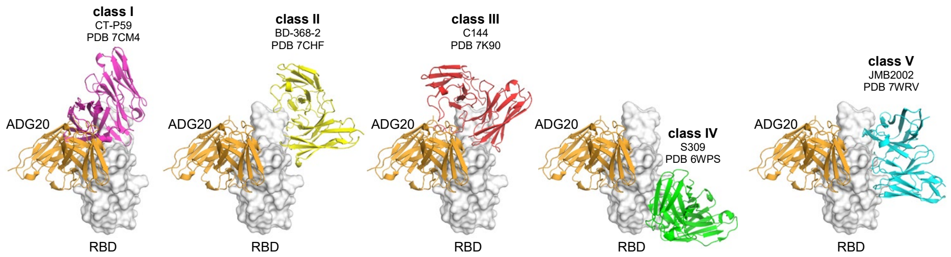

Fig. S1. The epitope of ADG20 is distinct from any of the antibody classes I-V analyzed in Yin et al. (1).

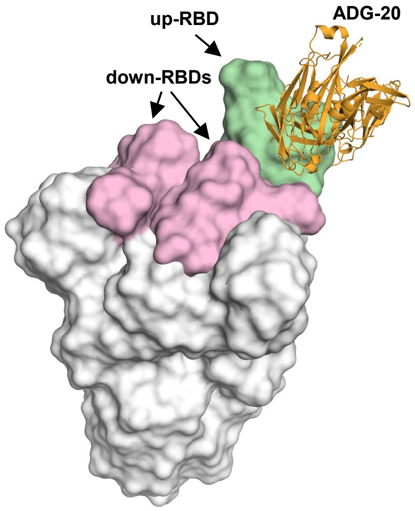

Fig. S2. Modelling the binding of ADG-20 on the homotrimeric spike (S) protein. The SARS-CoV-2 S trimer is shown with one RBD in the up conformation (green) and two RBDs in the down conformation (pink). PDB 7ND9 was used in the modeling (2). The complete epitope of ADG-20 is accessible only when the RBD is in the up, but not in the down, conformation.

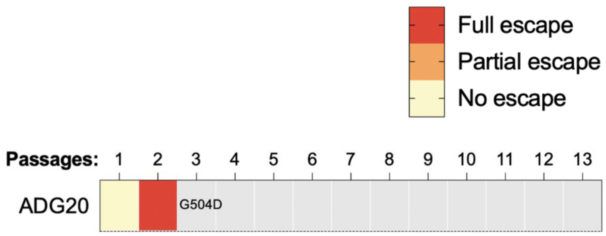

Fig. S3. An escape mutant of authentic SARS-CoV-2 against ADG-20. An in vitro SARS-CoV-2 G504D mutant escapes neutralization by ADG-20.

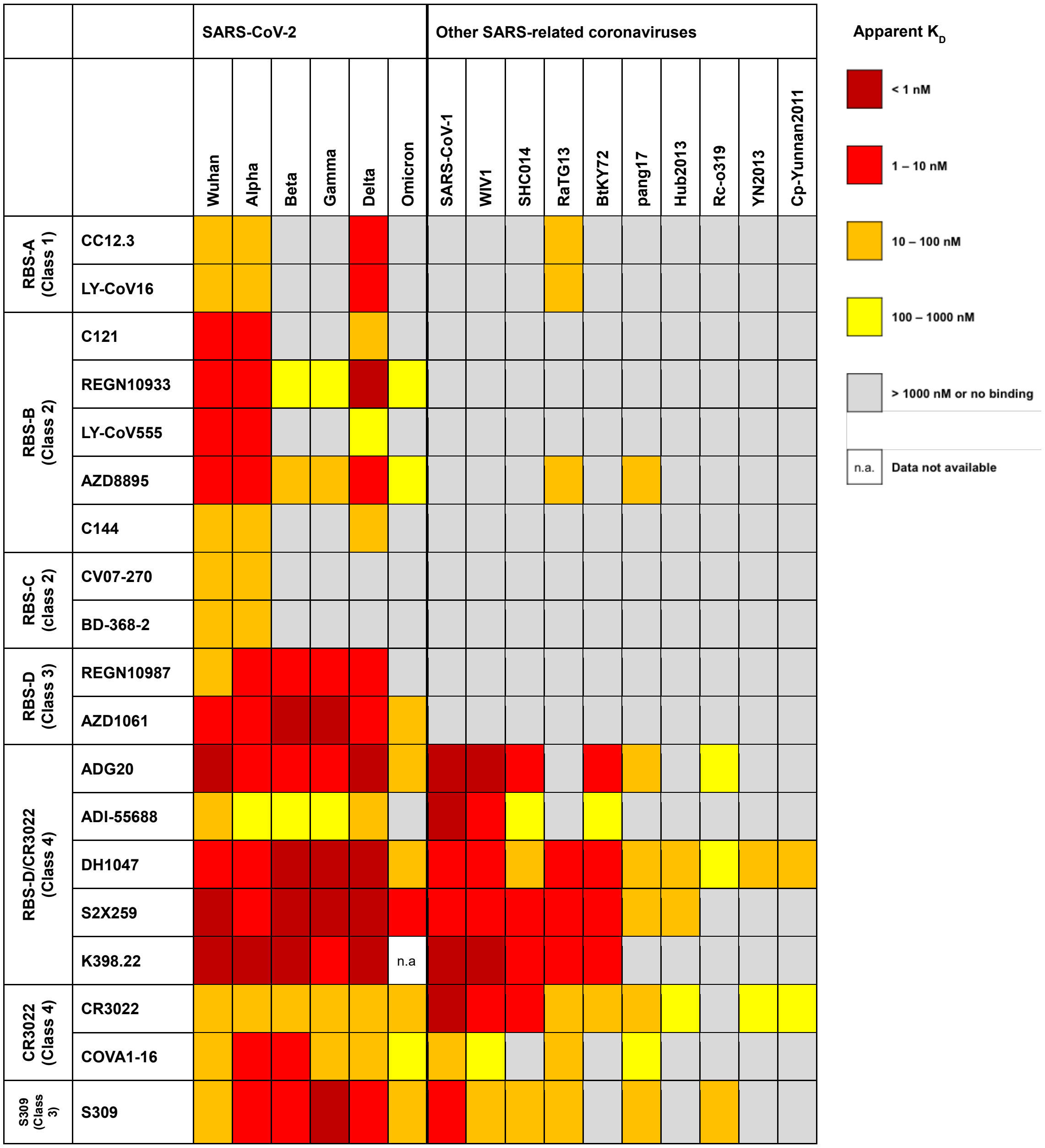
SARS-CoV-2
Other SARS-related coronaviruses
Wuhan
Alpha
Beta
Gamma
Delta
Omicron
SARS-CoV-1
WIV1
SHC014
RaTG13
BtKY72
pang17
Hub2013
Rc-o319
YN2013
Cp-Yunnan2011
RBS-A (Class 1)
CC12.3
LY-CoV16
RBS-B (Class 2)
C121
REGN10933
LY-CoV555
AZD8895
C144
RBS-C (class 2)
CV07-270
BD-368-2
RBS-D (Class 3)
REGN10987
AZD1061
RBS-D/CR3022 (Class 4)
ADG20
ADI-55688
DH1047
S2X259
K398.22
n.a
CR3022 (Class 4)
CR3022
COVA1-16
S309 (Class 3)
S309
Apparent K_D
< 1 nM
1 – 10 nM
10 – 100 nM
100 – 1000 nM
> 1000 nM or no binding
n.a.
Data not available

B

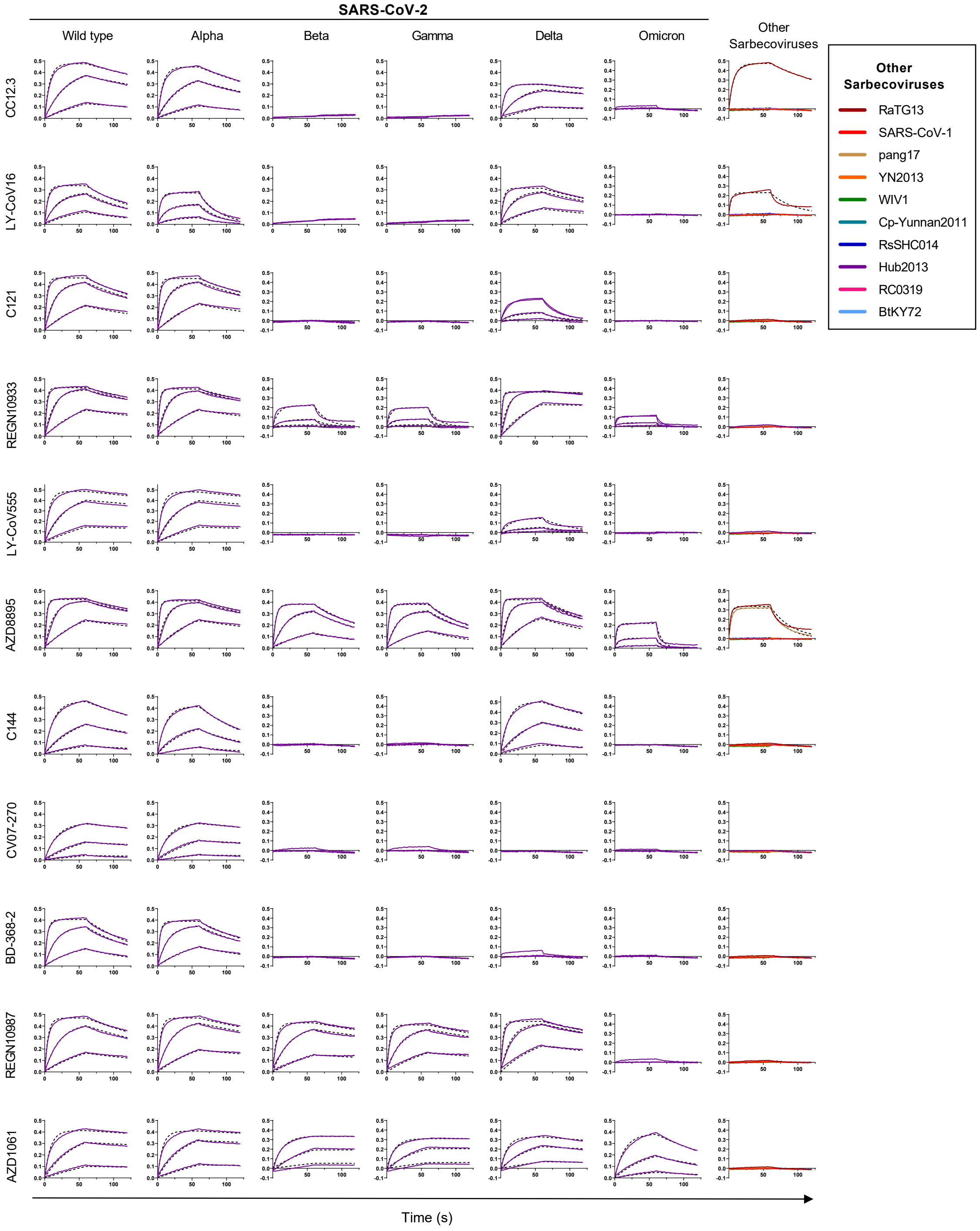
SARS-CoV-2
Wild type
Alpha
Beta
Gamma
Delta
Omicron
Other Sarbecoviruses
CC12.3
LY-CoV16
C121
REGN10933
LY-CoV555
AZD8895
C144
CV07-270
BD-368-2
REGN10987
AZD1061
Other Sarbecoviruses
RaTG13
SARS-CoV-1
pang17
YN2013
WIV1
Cp-Yunnan2011
RsSHC014
Hub2013
RC0319
BtKY72
Time (s)

C

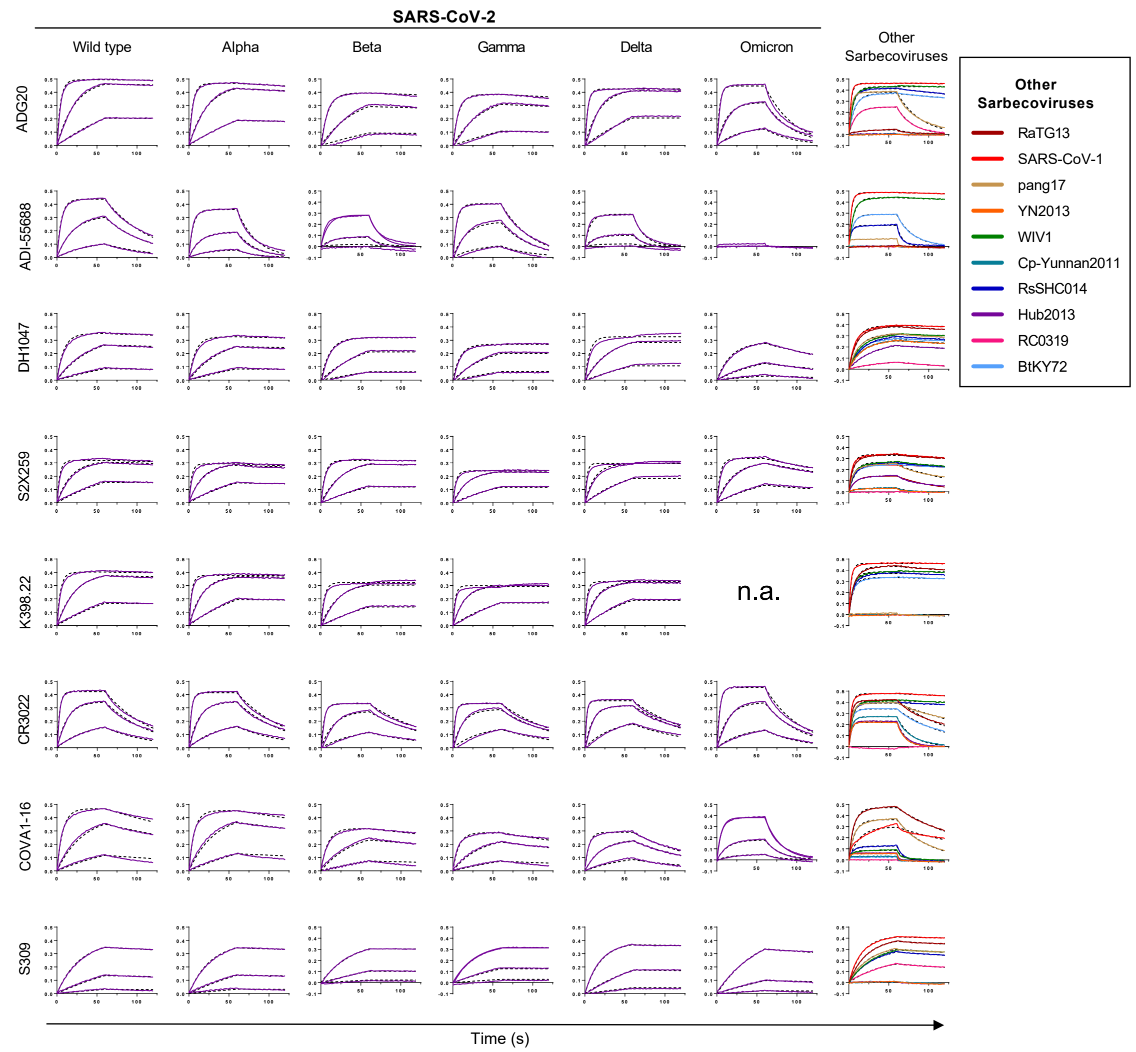

Fig. S4. Binding affinity of antibodies with coronavirus spike RBDs. (*A*) BioLayer interferometry (BLI) was used to assess the apparent K_D, where the IgGs were captured on the sensors and the RBD proteins flowed over. Epitope classification of the RBD antibodies that we previously defined (3) is shown in the left column, with the approximate corresponding classes defined in (4) shown in brackets. n.a. not available. (*B-C*) Sensorgrams for binding of IgGs and RBDs. The y-axis represents the response against different concentrations of RBD (x-axis in time, seconds). Solid lines represent the response curves and dashed lines represent the 1:1 binding model. The IgGs were loaded onto anti-human IgG Fc (AHC) biosensors and interacted with 5-fold

gradient dilution (500 nM – 20 nM) of SARS-CoV-2 RBDs, and 500 nM of RBDs of other sarbecoviruses.

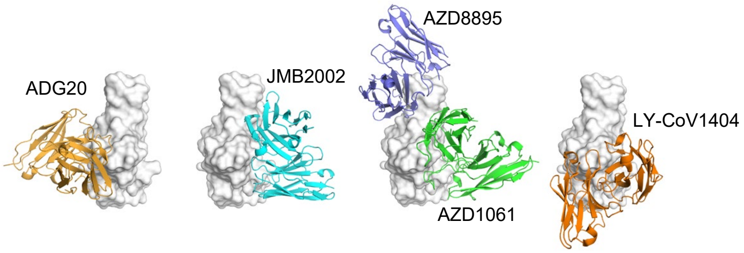

Fig. S5. Relative locations of ADG-20 and other RBD-targeting antibodies on the RBD. The structures of complexes JMB2002/RBD (PDB: 7WRV), AZD8895/AZD1061/RBD (7L7E), and LY-CoV1404/RBD (7MMO) were superimposed via their RBDs onto the structure of ADG20/RBD determined in this study. RBD is shown in a white surface, while antibodies are in cartoon representation. For clarity, only the variable domains of the antibodies are shown.

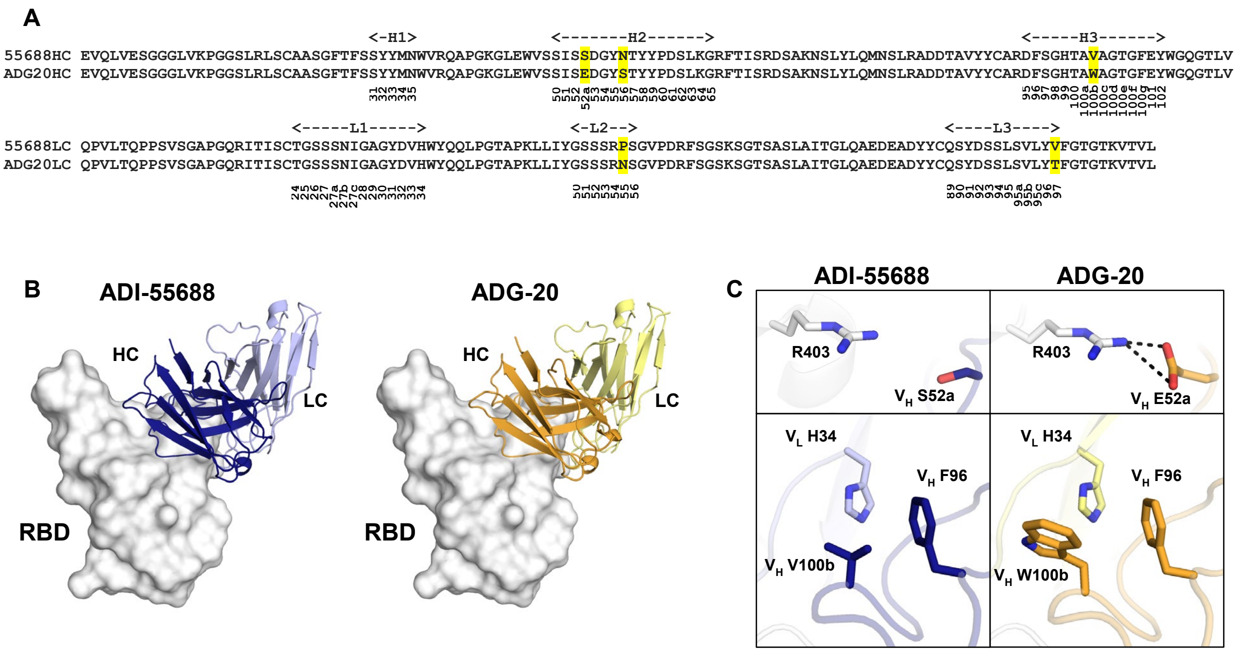

Fig. S6. Comparison between ADG-20 and its parent antibody ADI-55688. (*A*) Sequence alignment between ADG-20 and its parent antibody ADI-55688. Different residues are highlighted in yellow. Kabat numbering is shown under the CDR sequences. (*B*) Structural comparison between ADI-55688 and ADG-20. Both antibodies target the same epitope on SARS-CoV-2 RBD (white) in an identical binding mode. (*C*) Detailed interactions of the SARS-CoV-2 RBD with ADI-55688 and ADG-20. In B and C, heavy and light chains of ADI-55688 are shown in dark and light blue, and those of ADG-20 in orange and yellow. The RBD surface is shown in white. Hydrogen bonds are represented by black dashed lines.

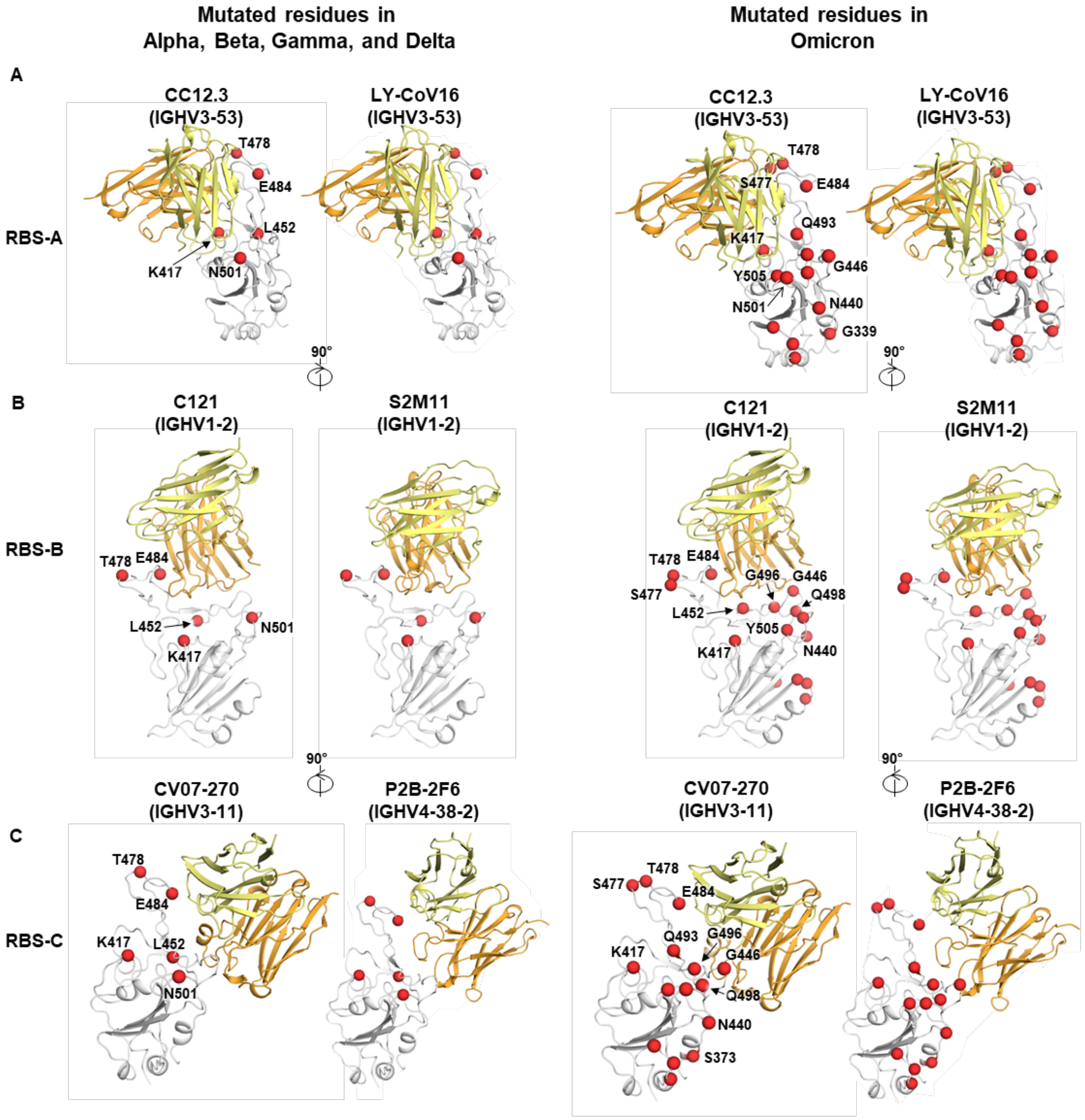

Fig. S7. Structures of representative antibodies targeting epitopes RBS-A, B, and C. SARS-CoV-2 RBD is represented by a white cartoon with mutated residues in VOCs shown as red spheres, where VOCs Alpha, Beta, Gamma, and Delta are shown in the left panels and Omicron in the right panels. Structures are shown in a same view in each panel. Labelling of some residues are omitted in the right panels for clarity. Heavy and light chains of the bound antibodies are shown in orange and yellow, respectively. Only the variable domains of the antibodies are shown. Structures used for the representative antibodies: CC12.3 (PDB: 6XC4), LY-CoV16/CB6 (7C01), C121 (7K8X), S2M11 (7K43), CV07-270 (6XKP), and P2B-2F6 (7BWJ).

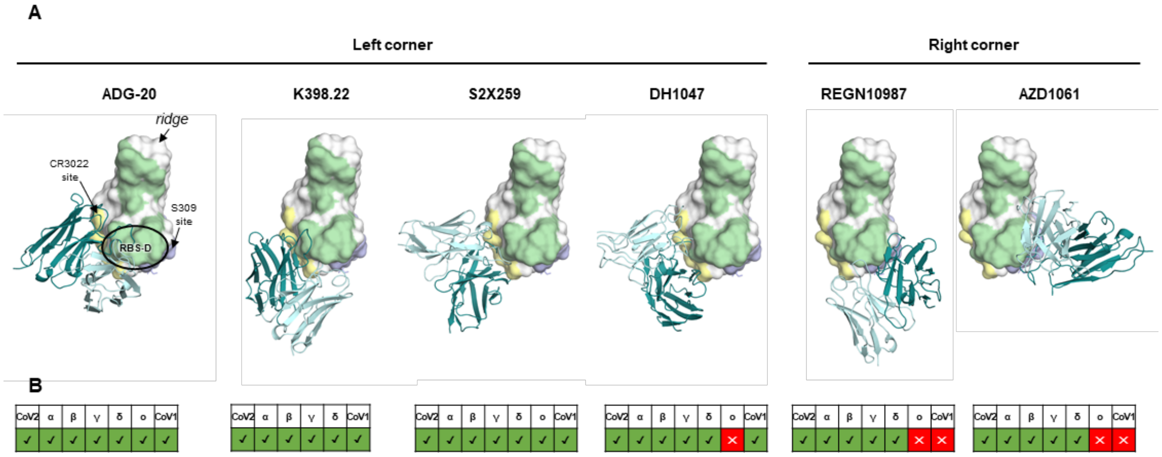

Fig. S8. Structural and functional comparison of antibodies targeting opposite corners of RBS-D. (*A*) Structures of RBS-D antibodies. SARS-CoV-2 RBD is shown as white surface with the RBS, CR3022 site and S309 site in green, yellow, and blue, respectively. Heavy and light chains of antibodies are shown as dark and light teal cartoons, respectively. The RBS-D region is highlighted with a black circle in the ADG-20 panel. All panels are in the same view. Structures used in this figure: ADG-20 (this study, PDB 7U2D), S2X259 (PDB 7RAL) (5), DH1047 (PDB 7LD1) (6), K398.22 (7), REGN10987 (PDB 6XDG) (8), AZD1061 (PDB 7L7E) (9). (*B*) Neutralization of each antibody against SARS-CoV-2 and VOCs, as well as SARS-CoV-1. Each panel corresponds to the complex immediately above in (*A*). Antibodies with detectable neutralization are shown in a green "✓" while a red "✕" represents no neutralization. Omicron data for K398.22 are not available.

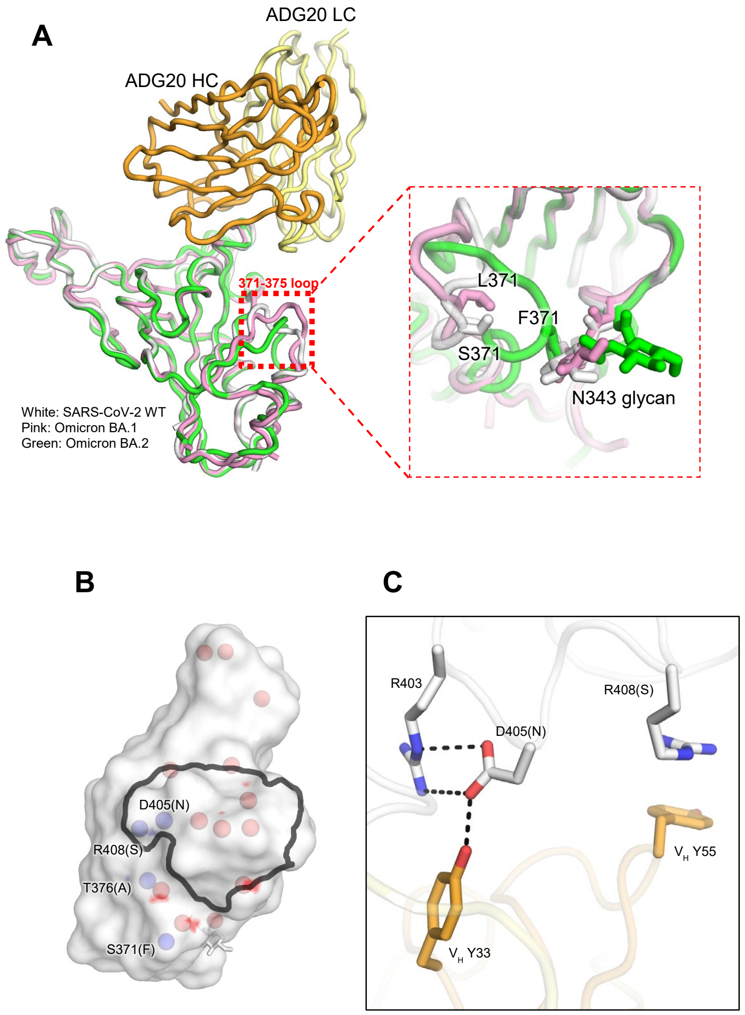

Fig. S9. Mutations of the Omicron BA.2 sub-lineage on the RBD. (*A*) Structural comparison between the ADG20-bound wild-type SARS-CoV-2 RBD, Omicron BA.1 and BA.2 RBDs. Crystal structure of ADG20 (heavy chain: orange, light chain: yellow) in complex with wild-type SARS-CoV-2 RBD (white) is from this study (PDB 7U2D). The superimposed Omicron BA.1 RBD (pink) and BA.2 RBD (green) are extracted from structures in PDBs 7QNW and 7UB5, respectively (10, 11). The 371-376 loop is highlighted in a red frame and has different backbone conformations in wild-type SARS-CoV-2, Omicron BA.1 (S371L, S373P, and S375F), and BA.2 (S371F, S373P, S375F, and T376A) The loop containing these mutations is also close to the glycan at N343. Changes in this region appear to affect ADG20 binding. (*B*) The RBD is represented by a transparent white surface. The ADG20 epitope is outlined. Mutated residues in the Omicron BA.2 variant are shown as red spheres, with those only in BA.2 but not BA.1 shown in blue. (*C*)

Detailed interactions between ADG20 (orange) and wild-type SARS-CoV-2 RBD (white). BA.2-specific mutations are shown in brackets in the labels. Mutation R408S may disrupt the cation-π bond formed with ADG20 V_H Y55, whereas D405N may retain hydrogen bonds with ADG20 V_H Y33 and RBD-R403 but lose a salt bridge with RBD-R403.

A

	S371	T376	D405	R408	G446	L452	F486	Q493
BA.1	L	-	-	-	S	-	-	R
BA.2	F	A	N	S	-	-	-	R
BA.2.12.1	F	A	N	S	-	Q	-	R
BA.4 & BA.5	F	A	N	S	-	R	V	-

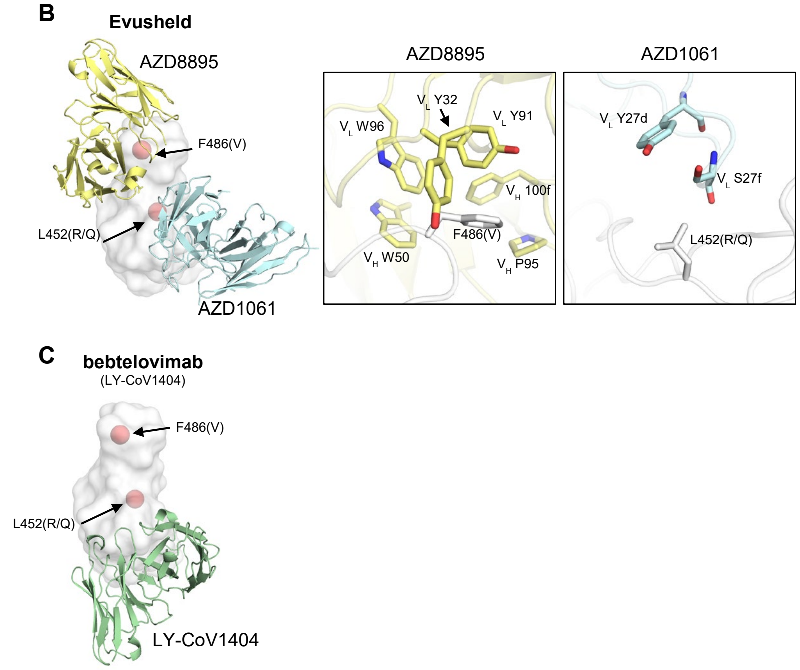

Fig. S10. Mutations in variants BA.2.12.1, BA.4, and BA.5 and their interactions with therapeutic antibodies. (*A*) Residues on the RBD of wild-type SARS-CoV-2 that differ in Omicron sub-lineages BA.1, BA.2, BA.2.12.1, BA.4, and BA.5 are indicated. Wild-type residues are shown in the first row, while residues in the Omicron variants are in the rows below. The same residues are represented by '-'. (*B-C*) Interactions between emerging mutations F486V and L452Q/R versus Evusheld (AZD8895 + AZD1061, PDB: 7L7E) and bebtelovimab (7MMO). Kabat numbering is assigned to all antibody residues.

Table S1. X-ray data collection and refinement statistics

**Data collection**	ADG-20 + SARS-CoV-2 RBD	ADI-55688 + SARS-CoV-2 RBD
Beamline	APS23ID-B	SSRL12-1
Wavelength (Å)	1.0332	0.9795
Space group	$P\ 4_1$	$P\ 4_1$
Unit cell parameters		
a, b, c (Å)	101.1, 101.1, 80.6	101.0, 101.0, 79.9
α, β, γ (°)	90, 90, 90	90, 90, 90
Resolution (Å) [a]	50.0-2.75 (2.81-2.75)	50.0-2.85 (2.90-2.85)
Unique reflections	20,542 (2064) [a]	18,826 (1811) [a]
Redundancy	11.2 (6.5)	8.9 (7.3)
Completeness (%)	97.6 (98.1)	100.0 (99.9)
$<I/\sigma_I>$	19.9 (1.0)	15.1 (1.0)
R_{sym}[b] (%)	11.4 (98.9)	14.5 (>100)
R_{pim}[b] (%)	3.4 (38.3)	5.1 (51.3)
$CC_{1/2}$[c] (%)	99.7 (69.3)	98.9 (59.0)
**Refinement statistics**		
Resolution (Å)	35.7-2.75	39.3-2.85
Reflections (work)	20,538	18,820
Reflections (test)	1,025	1,882
R_{cryst}[d] / R_{free}[e] (%)	21.9/25.8	25.4/28.4
No. of atoms	4,873	4,837
RBD	1,561	1,561
Fab	3,240	3,231
Ligands [f]	38	45
Solvent	34	0
Average *B*-values ($Å^2$)	72	66
RBD	69	67
Fab	73	65
Ligands	119	68
Solvent	56	N/A
Wilson *B*-value ($Å^2$)	72	70
**RMSD from ideal geometry**		
Bond length (Å)	0.002	0.002
Bond angle (°)	0.57	0.57
**Ramachandran statistics (%)** [g]		
Favored	96.1	96.1
Outliers	0.16	0.00
**PDB code**	7U2D	7U2E

[a] Numbers in parentheses throughout refer to the highest resolution shell.

[b] $R_{sym} = \Sigma_{hkl} \Sigma_i | I_{hkl,i} - <I_{hkl}> | / \Sigma_{hkl} \Sigma_i I_{hkl,i}$ and $R_{pim} = \Sigma_{hkl} (1/(n-1))^{1/2} \Sigma_i | I_{hkl,i} - <I_{hkl}> | / \Sigma_{hkl} \Sigma_i I_{hkl,i}$, where $I_{hkl,i}$ is the scaled intensity of the ith measurement of reflection h, k, l, $<I_{hkl}>$ is the average intensity for that reflection, and *n* is the redundancy.

[c] $CC_{1/2}$ = Pearson correlation coefficient between two random half datasets.

[d] $R_{cryst} = \Sigma_{hkl} | F_o - F_c | / \Sigma_{hkl} | F_o | \times 100$, where F_o and F_c are the observed and calculated structure factors, respectively.

[e] R_{free} was calculated as for R_{cryst}, but on a test set comprising 5% or 10% of the data excluded from refinement.

[f] Bound ligands are SO_4 and citrate.

[g] From MolProbity (12).

Table S2. Hydrogen bonds and salt bridges identified at the antibody-RBD interface using the PISA program.

ADG-20	Distance [Å]	SARS-CoV-2 RBD
**Hydrogen bonds**		
VH:SER56[OG]	3.8	ASP405[O]
VH:TYR33[OH]	2.7	ASP405[OD2]
VH:THR100E[OG1]	3.3	THR500[O]
VH:GLU52A[OE2]	3.7	ASN501[OD1]
VH:THR100[O]	3.0	GLN498[NE2]
VH:HIS99[O]	3.2	GLN498[NE2]
VH:ALA100A[O]	2.5	THR500[OG1]
VH:ALA100A[O]	3.7	ASN501[N]
VH:THR100[O]	3.5	ASN501[ND2]
VH:THR100E[OG1]	3.8	GLY502[N]
VH:ALA100C[O]	2.8	GLY502[N]
VH:GLU52A[OE2]	2.8	TYR505[OH]
**Salt bridges**		
VH:GLU52A[OE1]	3.8	ARG403[NH2]
VH:GLU52A[OE2]	2.5	ARG403[NH2]

ADI-55688	Distance [Å]	SARS-CoV-2 RBD
**Hydrogen bonds**		
VH:TYR33[OH]	2.8	ASP405[OD2]
VH:THR100E[OG1]	3.3	THR500[O]
VH:ALA100A[O]	2.4	THR500[OG1]
VH:ALA100A[O]	3.7	ASN501[N]
VH:THR100[O]	3.5	ASN501[ND2]
VH:ALA100A[O]	2.9	ASN501[ND2]
VH:ALA100C[O]	2.6	GLY502[N]
VH:TYR33[OH]	2.8	ASP405[OD2]
VH:THR100E[OG1]	3.3	THR500[O]
L:TYR31[OH]	2.7	GLN506[NE2]
L:TYR91[OH]	3.0	VAL503[N]

Table S3. Putative germline genes and CDR H3 sequences of antibodies targeting the RBS-D/CR3022 epitope

	IGHV	IGK(L)V	CDR H3 sequence	Reference
**ADG-20**	IGHV3-21	IGLV1-40	ARDFSGHTAWAGTGFEY	(13), this study
**DH1047**	IGHV1-46	IGKV4-1	ARDVRVDDSWSGYDLLSGGTYFDY	(6, 14)
**S2X259**	IGHV1-69	IGLV1-40	ARGFNGNYYGWGDDDAFDI	(5)
**K398.22**	Macaque IGHV3-73	Macaque IGLV2-32	TRVSIFGQFIVATYFDY	(7)

SI References

1. W. Yin *et al.*, Structures of the Omicron spike trimer with ACE2 and an anti-Omicron antibody. *Science* **375**, 1048-1053 (2022).

2. W. Dejnirattisai *et al.*, The antigenic anatomy of SARS-CoV-2 receptor binding domain. *Cell* **184**, 2183-2200.e22 (2021).

3. M. Yuan *et al.*, Structural and functional ramifications of antigenic drift in recent SARS-CoV-2 variants. *Science* **373**, 818-823 (2021).

4. C. O. Barnes *et al.*, SARS-CoV-2 neutralizing antibody structures inform therapeutic strategies. *Nature* **588**, 682–687 (2020).

5. M. A. Tortorici *et al.*, Broad sarbecovirus neutralization by a human monoclonal antibody. *Nature* **597**, 103-108 (2021).

6. D. Li *et al.*, In vitro and in vivo functions of SARS-CoV-2 infection-enhancing and neutralizing antibodies. *Cell* **184**, 4203-4219.e32 (2021).

7. W.-t. He *et al.*, Broadly neutralizing antibodies to SARS-related viruses can be readily induced in rhesus macaques. *bioRxiv* 10.1101/2021.07.05.451222 (2021).

8. J. Hansen *et al.*, Studies in humanized mice and convalescent humans yield a SARS-CoV-2 antibody cocktail. *Science* **369**, 1010-1014 (2020).

9. J. Dong *et al.*, Genetic and structural basis for SARS-CoV-2 variant neutralization by a two-antibody cocktail. *Nat. Microbiol.* **6**, 1233-1244 (2021).

10. W. Dejnirattisai *et al.*, SARS-CoV-2 Omicron-B.1.1.529 leads to widespread escape from neutralizing antibody responses. *Cell* **185**, 467-484.e15 (2022).

11. V. Stalls *et al.*, Cryo-EM structures of SARS-CoV-2 Omicron BA.2 spike. *bioRxiv* 10.1101/2022.04.07.487528 (2022).

12. V. B. Chen *et al.*, MolProbity: all-atom structure validation for macromolecular crystallography. *Acta Crystallogr. D Biol. Crystallogr.* **66**, 12-21 (2010).

13. C. G. Rappazzo *et al.*, Broad and potent activity against SARS-like viruses by an engineered human monoclonal antibody. *Science* **371**, 823-829 (2021).

14. D. R. Martinez *et al.*, A broadly cross-reactive antibody neutralizes and protects against sarbecovirus challenge in mice. *Sci. Transl. Med.* **14**, eabj7125 (2022).
